# Author Correction: The Hubble constant troubled by dark matter in non-standard cosmologies

**DOI:** 10.1038/s41598-022-26916-2

**Published:** 2023-01-05

**Authors:** J. S. Alcaniz, J. P. Neto, F. S. Queiroz, D. R. da Silva, R. Silva

**Affiliations:** 1 Observatório Nacional, General José Cristino 77, Rio de Janeiro, RJ 20921 400 Brasil; 2 Instituto de Física Gleb Wataghin, Universidade Estadual de Campinas, Campinas, SP 13083 859 Brasil; 3 International Institute of Physics, Universidade Federal Do Rio Grande Do Norte, Natal, RN 59078 970 Brasil; 4 Departamento de Física, Universidade Federal Do Rio Grande Do Norte, Natal, RN 59078-970 Brasil; 5 Millennium Institute for Subatomic Physics at High-Energy Frontier (SAPHIR), Fernandez Concha 700, Santiago, Chile; 6 Departamento de Fisica, Universidade Federal da Paraiba, João Pessoa, PB 58051 970 Brasil

Correction to: *Scientific Reports* 10.1038/s41598-022-24608-5, published online 22 November 2022

In the original version of this Article the author R. Silva was incorrectly affiliated with ‘Instituto de Física Gleb Wataghin, Universidade Estadual de Campinas, Campinas, SP, 13083-859, Brasil’ and ‘International Institute of Physics, Universidade Federal do Rio Grande do Norte, Natal, RN, 59078-970, Brasil’. The correct affiliation is listed below.

Departamento de Física, Universidade Federal do Rio Grande do Norte, Natal, RN, 59078-970, Brasil.

The original Article has been corrected.

